# Metabolomic Analysis of *Cricket paralysis* *virus* Infection in *Drosophila* S2 Cells Reveals Divergent Effects on Central Carbon Metabolism as Compared with Silkworm Bm5 Cells

**DOI:** 10.3390/v12040393

**Published:** 2020-04-01

**Authors:** Luo-Luo Wang, Luc Swevers, Lieven Van Meulebroek, Ivan Meeus, Lynn Vanhaecke, Guy Smagghe

**Affiliations:** 1 Guangdong Provincial Key Laboratory of Insect Developmental Biology and Applied Technology, Institute of Insect Science and Technology, School of Life Sciences, South China Normal University, Guangzhou 510631, China; Luoluo.Wang@scnu.edu.cn; 2 Department of Plants and Crops, Faculty of Bioscience Engineering, Ghent University, 9000 Ghent, Belgium; Ivan.Meeus@UGent.be; 3 Insect Molecular Genetics and Biotechnology, Institute of Biosciences and Applications, National Centre for Scientific Research “Demokritos”, 153 10 Athens, Greece; swevers@bio.demokritos.gr; 4 Laboratory of Chemical Analysis, Department of Veterinary Public Health and Food Safety, Ghent University, 9000 Ghent, Belgium; Lieven.Vanmeulenbroek@UGent.be (L.V.M.); Lynn.Vanhaecke@UGent.be (L.V.)

**Keywords:** metabolomics, persistent infection, host metabolism, *Cricket paralysis virus*, central carbon metabolism

## Abstract

High-throughput approaches have opened new opportunities for understanding biological processes such as persistent virus infections, which are widespread. However, the potential of persistent infections to develop towards pathogenesis remains to be investigated, particularly with respect to the role of host metabolism. To explore the interactions between cellular metabolism and persistent/pathogenic virus infection, we performed untargeted and targeted metabolomic analysis to examine the effects of *Cricket paralysis virus* (CrPV, *Dicistroviridae*) in persistently infected silkworm Bm5 cells and acutely infected *Drosophila* S2 cells. Our previous study (Viruses 2019, 11, 861) established that both glucose and glutamine levels significantly increased during the persistent period of CrPV infection of Bm5 cells, while they decreased steeply during the pathogenic stages. Strikingly, in this study, an almost opposite pattern in change of metabolites was observed during different stages of acute infection of S2 cells. More specifically, a significant decrease in amino acids and carbohydrates was observed prior to pathogenesis, while their abundance significantly increased again during pathogenesis. Our study illustrates the occurrence of diametrically opposite changes in central carbon mechanisms during CrPV infection of S2 and Bm5 cells that is possibly related to the type of infection (acute or persistent) that is triggered by the virus.

## 1. Introduction

Infections with cytopathic viruses typically cause cellular lethality, which explains their acute character, leading either to the death of the organism or clearing of the infection by the immune response. By contrast, non-cytopathic viruses can establish readily long-lasting (persistent) infections that manage the evasion of elimination by the immune system for very long periods [1]. Mechanisms to establish viral persistence not only include modulation of viral gene expression, subversion of apoptosis and immune evasion but can also involve the selection of particular cell types that are ideal for long-term preservation [2]. Although most studies have focused on alterations in the viral genome during establishment of persistent virus infections (and loss of the cytopathic character) in cell lines, e.g., [3,4], evidence exists for the importance of the interaction between the genetic make-up of both host cells and virus [5]. Host and cell tropism are considered very important characteristics of viruses and differences in efficiency of viral infection can be determined at each step of the viral replication cycle [6]. However, because of the importance of cellular entry in viral infection, viral tropism is usually distinguished between receptor-dependent and -independent (i.e., intracellular) mechanisms.

*Cricket paralysis virus* (CrPV) is a picorna-like virus with a (+)-ssRNA genome that has been used as a model for the study of antiviral resistance mechanisms in *Drosophila*, which includes inhibition of RNAi [7,8]. CrPV causes acute pathogenic infections in flies and *Drosophila* cell lines, which is related to the production of the viral 1A protein, a strong RNAi inhibitor [9].

In our previous study, it was observed that, when applied at relatively low MOI (multiplicity of infection), CrPV does not trigger an acute infection in silkworm Bm5 cells and other lepidopteran and coleopteran cell lines [10]. A period of viral persistence was observed during which the host cells maintained a normal growth rate and low levels of CrPV replication were detected. Only 2–3 weeks after virus inoculation, cytopathic effects started to emerge, which was accompanied with the transcriptional induction of immune pathway genes [10]. The slow kinetics of CrPV infection in Bm5 cells and the other lepidopteran and coleopteran cell lines [10] stood in strong contrast with the fast infection dynamics observed in *Drosophila* S2 cells. When inoculated at similar MOI as for Bm5 cells, infection of S2 cells became pathogenic already after 36–48 h, which was followed by widespread mortality [10]. Our observations therefore indicate important differences between S2 cells and other (coleopteran and lepidopteran) cell lines that were investigated with respect to the pathogenicity of CrPV infection. While acute infections can be triggered in lepidopteran Hi5 cells, a much higher MOI is needed than for S2 cells and kinetics remains much slower [11]. Injection of CrPV virions in insect species of three different orders (Lepidoptera, Hymenoptera and Orthoptera) also did not trigger pathogenic infections [12]. Thus, S2 cells seem to represent a unique case with respect to sensitivity to CrPV infection that stands in stark contrast with infections in other cell lines. From this point of view, the examination of the changes in the metabolome in response to CrPV infection in S2 cells is of particular interest since it would be representative of a cell type that is very sensitive to CrPV infection. The comparison with the persistent condition in Bm5 cells furthermore could reveal important insights regarding the impact of the metabolome on the type of CrPV infection (persistent or acute) that will occur.

In this study, untargeted and targeted metabolomics was applied to S2 cells at different stages of acute infection with CrPV (6 h, 12 h, 24 h and 36 h). The samples of acute infection of S2 cells were analyzed together with samples of persistent infection of Bm5 cells [13], to allow direct comparison of metabolic changes in the different cell lines. The metabolomics approach revealed striking differences with respect to central carbon metabolism during the course of infection in the two cell lines. While carbohydrates and amino acids increased in abundance during the persistent phase of infection in Bm5 cells, the metabolites became depleted during the early and middle stages of acute infection in S2 cells. At late stages of infection (i.e., 2–3 weeks in Bm5 cells, while 24–36 h in S2 cells), an opposite pattern was observed (i.e., decrease in Bm5 and increase in S2). Central carbon mechanism is therefore proposed to represent an important intrinsic host factor that affects the outcome of CrPV infection and that can be subject to manipulation by CrPV for optimal replication. Those findings may have implications for the manipulation of viral pathogenesis and the development of persistently infected viral culture systems.

## 2. Materials and Methods

### 2.1. Cell Culture and Virus Infection

S2 cells were maintained at 27 °C in Insect-Xpress Medium (VWR International) supplemented with 10% fetal bovine serum (FBS). The CrPV suspension that was used to infect S2 cells was identical to the one used to infect Bm5 cells [13]. S2 cells were seeded at 1 × 10^6^ cells per mL in a 12-well plate followed by inoculation with CrPV at MOI 1.

### 2.2. Experimental Design

**Figure 1:**
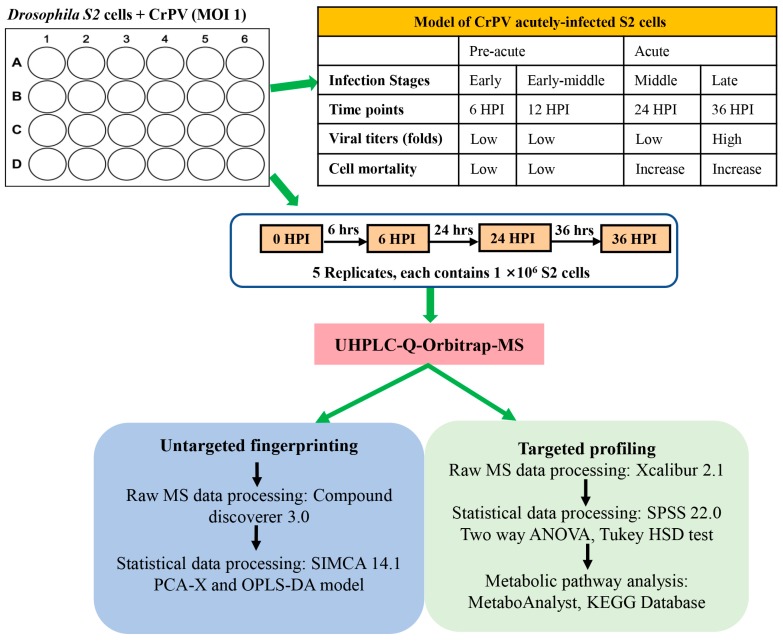
Experimental design and work flow of the metabolomics study. S2 cells were infected with *Cricket paralysis virus* (CrPV) at MOI (multiplicity of infection) 1 and cellular extracts (5 replicates) were prepared at different times after infection. Cellular extracts were processed by ultra-high performance liquid chromatography hyphenated to quadrupole-orbitrap high-resolution mass spectrometry (UHPLC-Orbitrap-MS) for both untargeted and targeted metabolomics. In untargeted metabolomics (or fingerprinting), all possible metabolites that were differentially present in experimental samples were determined by principal component analysis (PCA-X) and orthogonal partial least square-discriminant analysis (OPLS-DA) modeling. In targeted metabolomics (or profiling), the differential presence of a collection of approximately 300 known metabolites among experimental samples was determined directly. Pathway analysis was performed on identified metabolites that significantly changed in abundance among different experimental samples.

As illustrated in Figure 1, for infected S2 cell cultures, samples (*N* = 5) were collected at four time points during which infection rapidly becomes pathogenic (6–36 h post infection; 6 HPI, 12 HPI, 24 HPI and 36 HPI, respectively) [10]. Cellular extracts before CrPV infection (0 HPI) were also prepared as control samples. At each sampling time point, one well of the 12-well plate was used for quantification of cell numbers. Consequently, the same number of cells (10^6^) was used for metabolite extraction at each time point.

### 2.3. Extraction of Intracellular Metabolites and Ultra-High Performance Liquid Chromatography Hyphenated to Quadrupole-Orbitrap High-Resolution Mass Spectrometry (UHPLC-Q-Orbitrap-HRMS) Analysis

Preparation of S2 cell samples for metabolome analysis was carried out using a protocol that was identical to the one applied for Bm5 cells [13]. One milliliter of ice-cold 50:50 (v:v) methanol:water mixture was used to extract mainly polar metabolites. After sonication, the cellular debris was removed by centrifugation, the supernatant dried and subsequently re-suspended in 100 μL of ultrapure water before analysis [13]. Valine-d_8_ (27 μL, 25 ng/μL; Sigma-Aldrich) was added as internal standard (ISTD) to each sample.

Chromatographic separation was achieved using a Dionex Ultimate 3000 XRS ultra-high performance liquid chromatography (UHPLC) system (Thermo Fisher Scientific), which was equipped with an Acquity UPLC HSS T3 column (1.8 μm, 150 mm × 2.1 mm) (Waters). The binary solvent system consisted of ultrapure water and acetonitrile, and a gradient program according to De Paepe et al. [14] was applied. The solvent flow rate was 400 μL/min and the column oven temperature was 40 °C. Analyzed samples corresponded to 100 μL of cellular extract. Mass analysis was performed on a Q-Exactive^TM^ Orbitrap mass analyzer (Thermo Fisher Scientific) that was equipped with a HESI-II source, operating in polarity switching mode. Instrumental settings were identical as described in De Paepe et al. [14]. Quality control (QC) samples for data normalization and instrument conditioning were prepared as a pool of all extracts. Experimental samples were run in a randomized order, except for QC samples, which were analyzed in duplicate after every ten experimental samples.

### 2.4. Data Analysis

#### 2.4.1. Untargeted Data Analysis

The Sieve 2.1 software package (Thermo Fisher Scientific) was used for peak extraction and alignment, deconvolution and noise removal. Similarly to the analysis of the samples of CrPV-infected Bm5 cells [13], data for positive and negative ionization mode were analyzed separately and identical parameter settings for component characterization were used [13]. The relative abundance of each component in each sample was calculated after normalization using the total ion count (TIC) and the corresponding mean abundance of the two internal QC samples [14,15]. Unsupervised principal component analysis (PCA) and supervised orthogonal partial least square-discriminant analysis (OPLS-DA) were performed using SIMCA^TM^ 13.5.0 (Umetrics). CV-ANOVA (*p*-value < 0.05), permutation testing (*N* = 20), Q^2^ (>0.5) and R^2^Y (>0.5) [14] were used as quality parameters for model validation (after log-transformation and Pareto-scaling of the data).

#### 2.4.2. Targeted Data Analysis

The detection and determination of the relative abundances of 297 metabolites was carried out using Xcalibur^TM^ 2.1 (Thermo Fisher Scientific). Identification of the metabolites was based on an in-house library of analytical reference standards, taking into account the accurate mass (*m/*z-value) of the molecular ion, the relative retention time (with respect to the internal standard) and the ^13^C/^12^C isotope ratio. Two-way ANOVA and Tukey HSD test was performed using SPSS 22.0 **(**IBM), and TIC/QC normalized data were plotted using GraphPad Prism v.400 (Graphpad Software). MetaboAnalyst 4.0 (http://www.metaboanalyst.ca/) was employed for metabolite pathway construction.

## 3. Results

A previous study by our group revealed that pathogenic infection of lepidopteran Bm5 cells and dipteran S2 cells with CrPV occurred with different kinetics [11]. While in S2 cells cytopathic effects became evident already after 36–48 h, pathogenicity in Bm5 cells was observed only after 2–3 weeks. A follow-up study described the changes in the metabolome during the persistence period in Bm5 cells (2 days, 1 week and 2 weeks post-infection) until the transition to pathogenesis (3 weeks post-infection) [13]. In the present study, we describe the application of untargeted and targeted metabolomics to different stages of acute infection of S2 cells with CrPV, for which samples at comparable levels of virus replication were collected (prior to pathogenesis: 6 HPI, 12 HPI; transition to pathogenesis: 24 HPI, 36 HPI; Figure 1).

### 3.1. Global Metabolic Changes in S2 Cells Following CrPV Infection

**Figure 2:**
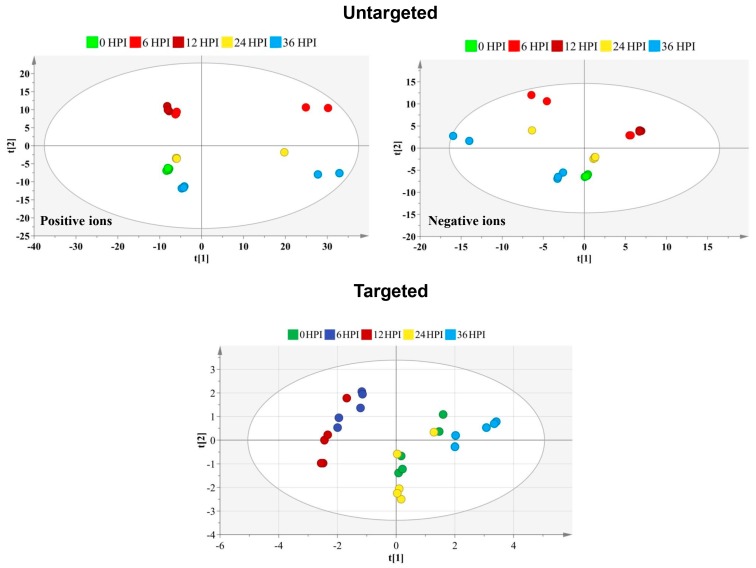
Score plot generated from the PCA-X model of untargeted and targeted metabolites. For the untargeted approach, the principal components analysis (PCA) score plot is presented separately for the positive and negative ion mode. The t [1] and t [2] values of each data point are the average scores of all samples in the principal components 1 and 2 of the model, respectively. Below is displayed the PCA score plot based on the 59 targeted metabolites identified at different time points after CrPV infection in S2 cells. Samples of different time points are shown in different colors that reveal satisfactory separation among particular samples. Incomplete separation is observed between 6 HPI and 12 HPI time points and between 0 HPI and 24 HPI samples.

An untargeted metabolomics approach was first performed to determine global changes in cellular metabolism during CrPV infection of S2 cells. Sieve^TM^ 2.1 data preprocessing resulted in 2079 (positive ionization mode) and 689 (negative ionization mode) features in S2 cells. Although PCA-X score plots initially did not reveal tightly clustered groups corresponding to the different time points of CrPV infection (Figure 2), subsequent OPLS-DA analysis could clearly differentiate the different experimental samples when making the comparison between each post-infection time point and the control group (Table 1).

### 3.2. Overview of Targeted Metabolite Profiling in S2 Cells Following CrPV Infection

Among approximately 300 polar metabolites that were evaluated, 59 metabolites were identified and used for semiquantitative metabolic profiling of S2 cells. These metabolites included 32 amino acids, 13 carbohydrates, 7 carboxylic acids and 7 compounds from other chemical classes (Table S1). Using metabolite profiles, the PCA-X score plot revealed incomplete separation of samples at the different time points (Figure 2). More specifically, 6 HPI and 12 HPI samples were grouped together while also 0 HPI and 24 HPI samples overlapped, reflecting more limited changes in metabolite abundance as for infection of Bm5 cells.

### 3.3. Distinct Metabolome Changes Triggered by CrPV Infection in S2 Cells

**Figure 3:**
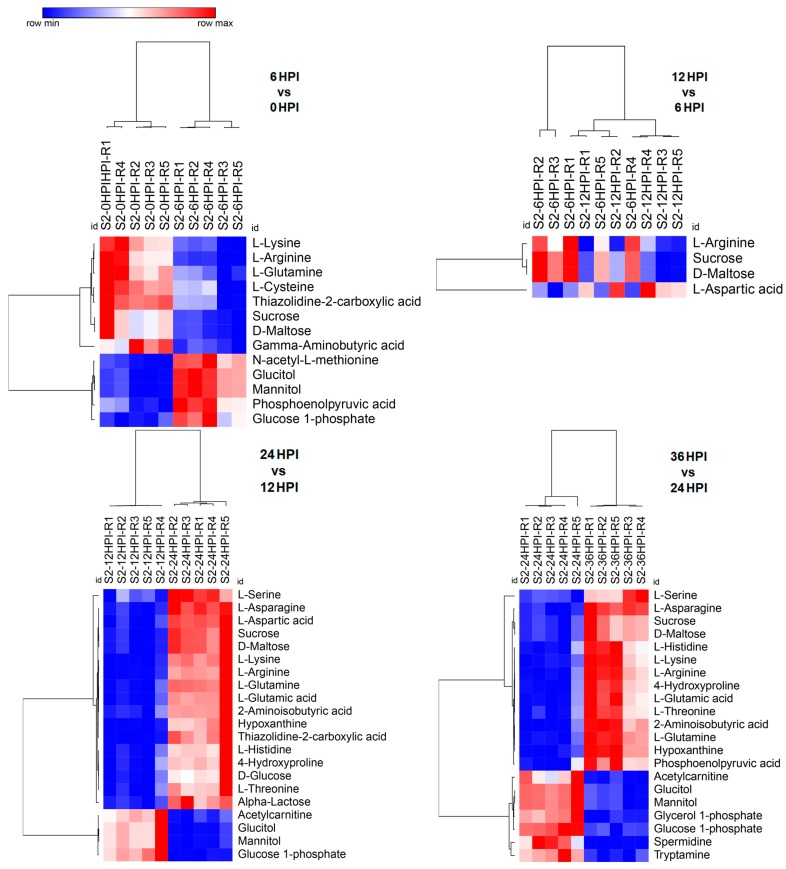
Heat maps of abundance of metabolites at different time points after CrPV infection: comparison between consecutive stages of infection. Hierarchical clustering (one minus Pearson correlation) was used to separate individual samples (X-axis). The Y-axis represents individual metabolites that were identified and showed changes in abundance with respect to preceding and following time points (*p* < 0.05). Normalized signal intensities are visualized as a color spectrum in the heat maps. Red and blue represent high and low expression, respectively, of metabolites.

In CrPV-infected S2 cells a higher proportion of metabolites decreased in abundance at early time points (6 HPI and 12 HPI) and at late stages (24 HPI and 36 HPI) a prominent increase was detected as compared to uninfected (0 HPI) cells (Figure S1). When consecutive stages of infection in S2 cells were compared, limited changes in metabolite abundance were observed during early stages that were biased towards a decrease in abundance (transition from 0 to 6 HPI and from 6 to 12 HPI; Figure 3). At late stages, however, an increase in a considerably higher number of metabolites was evident (transition from 12 to 24 HPI and from 24 to 36 HPI; Figure 3). It was also noted that relatively few differences were observed between uninfected cells and the 24 HPI sample (Figure 2 and Figure S1).

Metabolite pathway analysis showed that aminoacyl-tRNA biosynthesis was the most commonly altered pathway during CrPV infection of S2 cells (Table 2). Also the involvement of carbohydrate metabolism pathways (“Galactose metabolism” and “Starch and sucrose metabolism”; Table 2) was observed.

### 3.4. Abundance Analysis of Carbohydrates and Amino Acids during host-CrPV Infection

**Figure 4:**
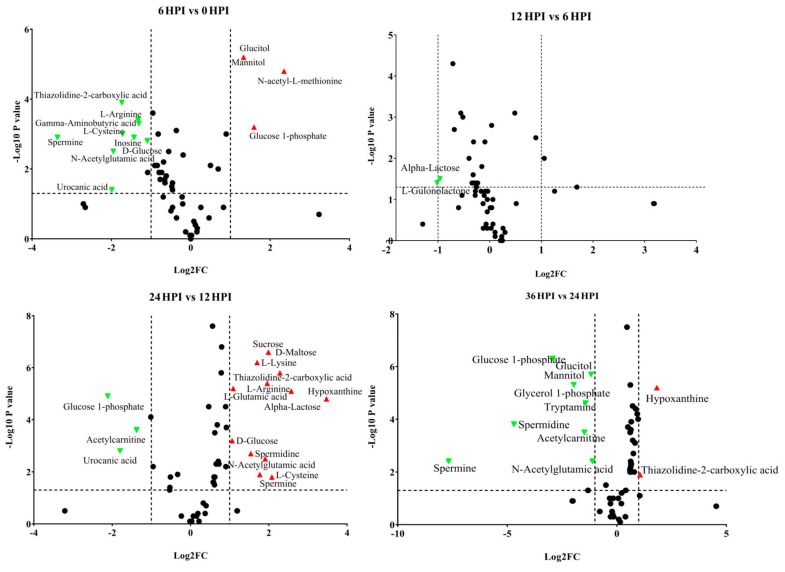
Volcano plots of all identified metabolites in S2 cells at different time points of CrPV infection: comparison between consecutive stages of infection. In the volcano plot, the log scaled value of the fold change (FC, x-axis) is plotted against the –log 10 *p*-value from multiple *t*-test analysis (y-axis). The dashed lines delineate metabolites with fold change > 2 and *p*-value < 0.05. Compound identities are displayed for both upregulated (right side; red upward triangle) or downregulated (left side; green downward triangle) metabolites.

Depending on the particular time point, 10.3%–41.4% of detected metabolites were significantly changed in S2 cells during the course of CrPV infection. Volcano plots were generated to identify the most significantly changed metabolites at different time points (Figure 4). Significant changes in abundance of amino acids, carbohydrates and other metabolites were observed in CrPV-infected S2 cells (Figure 4 and Figure S2; also summarized in Table 3 and Table S1).

**Figure 5:**
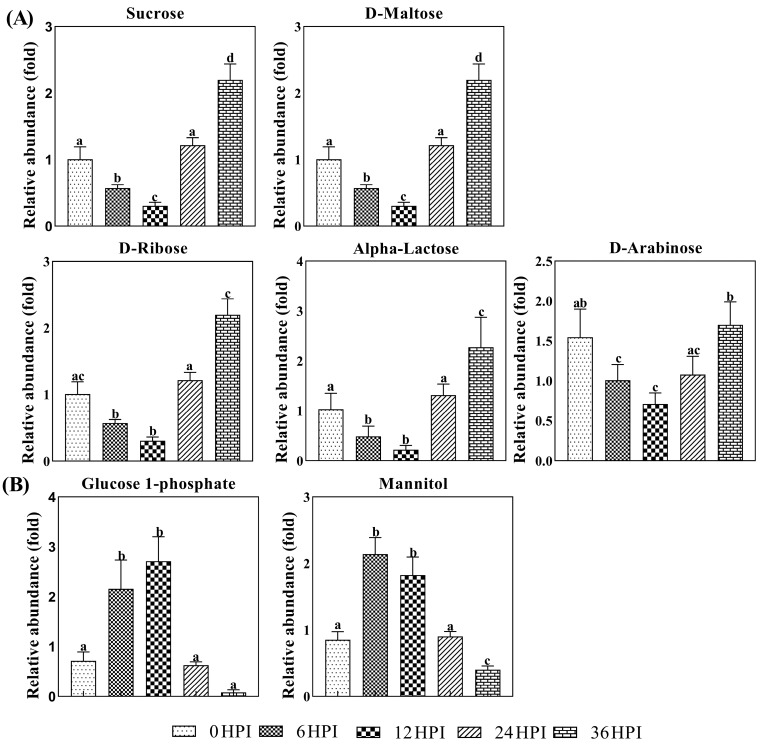
Abundance analysis of carbohydrates identified in S2 cells from different CrPV infection stages. The data shown are mean ± SE (*n* = 5). Different letters indicate significant differences (*p* < 0.05, one-way ANOVA followed by Tukey’s HST test).

A more detailed analysis of changes in the levels of carbohydrates in S2 cells is presented in Figure 5. Carbohydrates such as sucrose, arabinose, ribose, maltose and alpha-lactose declined during the early infection stages before increasing again at the pathogenic stage. On the other hand, levels of mannitol/glucitol and glucose-1-phosphate were highest at the 6 HPI and 12 HPI stages (Figure 5; Table S1).

**Figure 6:**
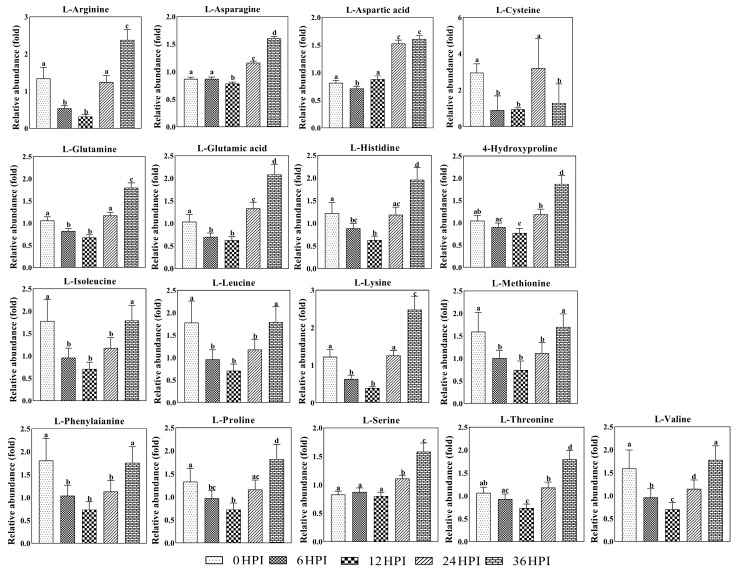
Abundance analysis of amino acids identified in S2 cells from different CrPV infection stages. The data shown are mean ± SE (*n* = 5). Different letters indicate significant differences (*p* < 0.05, one-way ANOVA followed by Tukey’s HST test).

Next, analysis was performed on the abundance of 17 key amino acids (Figure 6). As was the case for most carbohydrates, amino acids typically declined during early stages (6 HPI and 12 HPI) and increased during later stages (24 HPI and 36 HPI). For a few amino acids (aspartic acid, asparagine and serine), only the increase during the late stages was observed (Figure 6).

### 3.5. Key Metabolites Required for Acute Infection of CrPV in S2 Cells

**Figure 7:**
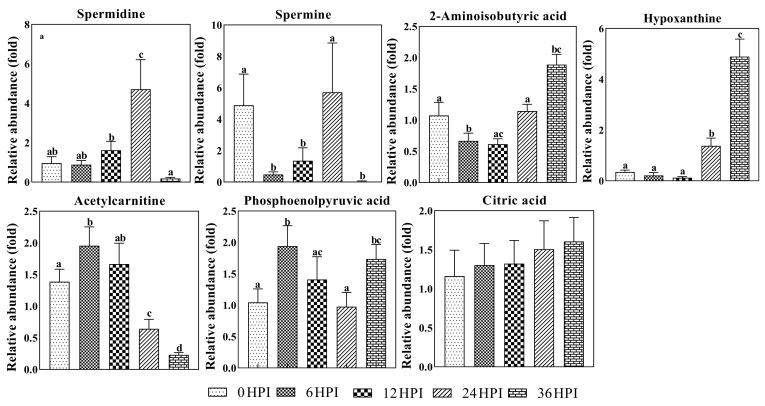
Abundance analysis of selected metabolites identified in S2 cells from different CrPV infection stages. The data shown are mean ± SE (*n* = 5). Different letters indicate significant differences (*p* < 0.05, one-way ANOVA followed by Tukey’s HST test).

Spermine and spermidine are polycations that play important roles during viral replication and translation [16,17]. As was also observed in Bm5 cells, dynamic changes in levels of both polyamines were found during CrPV infection in S2 cells (Figure 7). During early stages (6 HPI and 12 HPI), levels of both polyamines were low, with spermine showing a drop in abundance compared to uninfected cells. At 24 HPI, however, a large increase was observed, before a precipitous decline at 36 HPI (Figure 7).

Several other metabolites also show significant changes during CrPV infection in S2 cells. Levels of hypoxanthine, a purine derivative and intermediate in the metabolism of nucleic acids, increased more than 10-fold during the pathogenic stages of infection (Figure 7). Additionally, 2-amino-isobutyric acid, an end-product of pyrimidine metabolism, increased in abundance at 36 HPI. Acetylcarnitine, which plays a role in mitochondria energy production [18], on the other hand, decreased as infection progressed (Figure 7). By contrast, other molecules involved in the generation of energy, such as phosphoenolpyruvate and citric acid, did not show important changes during CrPV infection.

### 3.6. Comparison of Metabolite Changes between Persistent Infection in Bm5 Cells and Acute Infection in S2 Cells

Untargeted metabolomics was able to detect a larger number of ions in S2 cells than in Bm5 cells (2079 and 689 versus 1193 and 560 in positive and negative ionization mode, respectively). In Bm5 cells, PCA-X analysis generally resulted in clear separation of the samples at different time points [13], while considerable overlap among samples was observed for CrPV infection of S2 cells (Figure 2). OPLS-DA analysis, on the other hand, revealed that the metabolome also changed considerably as CrPV infection proceeds in S2 cells (Table 1), indicating that metabolites exist that significantly changed in abundance at different time points. As pointed out before [13], the identification of such compounds in future experiments should reveal additional important insights regarding the regulation of the progression of viral infection by cellular metabolism.

Among the approximately 300 polar to medium-polar metabolites that were examined in the targeted approach, almost identical metabolites were identified in both Bm5 cells and S2 cells. Among the 59 metabolites (31-32 amino-acids, 13 carbohydrates, 7 carboxylic acids and 7–8 compounds from other chemical classes) that were identified, 57 metabolites were quantified in all the samples from Bm5 and S2 cells. The metabolites cadaverine and allantoin were only identified in Bm5 cells; conversely, inosine and tryptamine were only detected in S2 cells.

More dramatic changes were found in Bm5 cells as compared to S2 cells during the course of CrPV infection (Table 3). On one hand, in Bm5 cells, CrPV infection resulted in significant changes of 34 (58%), 45 (76%), 42 (71%) and 43 (73%) metabolites in the 2 DPI, 1 WPI, 2 WPI and 3 WPI samples, respectively, as compared to the control group 0 HPI [13]. In comparison, much milder changes were observed in CrPV-infected S2 cells, where only 13 (22%), 21 (36%), 6 (10%) and 24 (41%) of the detected metabolites were significantly changed at 6 HPI, 12 HPI, 24 HPI and 36 HPI, respectively (with respect to uninfected cells; *p* < 0.05).

Metabolite pathway analysis identified amino acid synthesis and metabolism as the major metabolic pathways that were significantly altered during CrPV infection in both Bm5 and S2 cells (Table 2; [13]). While in Bm5 cells “glutathione metabolism” was also considered important at 3 WPI, more emphasis was detected on “galactose metabolism” at 12 HPI and 24 HPI and on “starch and sucrose metabolism” at 12 HPI in S2 cells (Table 2).

**Figure 8:**
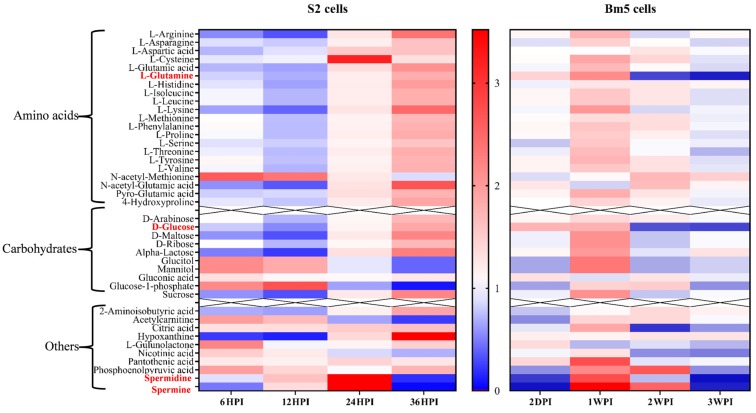
Comparison of relative abundances of metabolites after infection of S2 and Bm5 cells with CrPV. Intensities reflect relative decrease (in blue) or increase (in red) of the metabolite with respect to uninfected cells (average of *N* = 5). The different pattern of amino acids and carbohydrates in S2 cells (down-up) and Bm5 cells (up-down) is clearly apparent. Red colored are glutamine and glucose that show a characteristic increase at the end of infection with the S2 cells, while there was an opposite effect with a clear unique decline in Bm5 cells. Spermine and spermidine that show a dynamic pattern in both types of infections are also indicated.

However, while amino acids were considered the metabolites with the greatest significance during the two types of infection, striking differences exist in their pattern of change between Bm5 and S2 cells (Figure 8). While in Bm5 cells carbohydrates and amino acids increased in abundance during CrPV infection, particularly at 1 WPI, the same metabolites decreased during early infection (6 HPI and 12 HPI) in S2 cells before they increased again (24 HPI and 36 HPI; Figure 8). In contrast to S2 cells, metabolite levels decreased again during late infection in Bm5 cells although they generally remained at higher levels than in uninfected cells (with glutamine and glucose as notable exceptions; Figure 8). Thus an inverted pattern of changes in the levels of amino acids and the main carbohydrates was observed between the two cell lines: up-and-down in Bm5 cells, while down-and-up in S2 cells (Figure 8).

Three carbohydrates, i.e., glucitol, mannitol (involved in osmoregulation; [19,20]) and glucose-1-phosphate (that functions as a precursor of UDP-glucose that has a central role in glycogen and glycoprotein synthesis; [21]), exhibited similar kinetics in Bm5 and S2 cells, characterized by an increase in middle and a decrease in late stages (Figure 8).

The pattern in changes in polyamines was also rather similar between Bm5 and S2 cells, with a drop during early infection (except spermidine in S2 cells), an increase during middle stages and a drop at late infection (Figure 8). Increase in polyamines therefore may be associated with stimulation of viral replication (transition to pathogenicity) while their depletion occurs during advanced pathogenesis and cell mortality.

During infection of Bm5 cells, metabolites associated with energy production and biosynthesis, such as citric acid and phosphoenolpyruvate, followed a dynamic pattern that is similar to most carbohydrates and amino acids. In S2 cells, on the other hand, no changes in citric acid were observed while rather mild fluctuations in abundance were observed for phosphoenolpyruvate (Figure 8). On the other hand, the levels of 2-amino-isobutyric acid, an end-product of pyrimidine metabolism, accumulated during late phases of infection in both Bm5 and S2 cells, while this metabolite also showed a characteristic drop during middle infection in S2 cells (Figure 8).

Unique features in CrPV infection of Bm5 cells also included changes in vitamin-related metabolites (pantotheinic acid, nicotinic acid and L-gulunolactone), a product of purine metabolism (allantoin) and the diamine cadaverine (Figure 8). In S2 cells, the high levels of hypoxanthine (nucleic acid metabolite) during the final stages and the decline of acetylcarnitine (with role in energy metabolism in mitochondria; Figure 8) are characteristic. Acylcarnitines play a role in the transport of fatty acyl-CoA from the cytoplasm to mitochondria for β-oxidation and energy production [22]. The decline of acetylcarnitine during late infection of S2 cells may be related to alterations in energy metabolism or exhaustion at the end of the infection cycle of CrPV in S2 cells.

## 4. Discussion

Since differences in the kinetics of CrPV infections could serve as a model for studying the parameters that maintain viral persistence versus those that cause pathogenicity, we are interested to investigate how specific metabolites would change in the course of infection and which changes would be characteristic for the transition from persistence to pathogenicity. More specifically, host cell differences can become apparent after comparison of the metabolome between Bm5 and S2 cells that may be able to explain the dissimilar infection kinetics between the two cell lines. The persistent mode of infection that is observed in Bm5 cells was also observed in nine lepidopteran and two coleopteran cell lines while acute infection at low MOI was a unique feature of S2 cells [10]. These observations indicate that persistent infections of CrPV are rather common and that the acute infection of S2 is a rarer phenomenon.

Our analysis revealed that the metabolic profile of Bm5 cells became much more dramatically modified at different stages during CrPV infection than the metabolome of S2 cells (see Table 3 for summary with respect to targeted metabolites). Moreover, different infection stages differed significantly in both Bm5 and S2 cells with respect to metabolite dynamics. Regarding the Bm5-CrPV infection model, it was expected that the metabolic needs for the phases of viral persistence (2 DPI and 1 WPI), the transition to pathogenesis with increased viral replication (2 WPI) and advanced pathogenesis (3 WPI) would be very different from each other [13]. In the first instance, similar stages could be expected during CrPV infection of S2 cells, albeit on a shorter time scale (36 h versus 3 weeks; Figure 1). Considering the distinct metabolic effects at different stages of CrPV infection, it was of interest to examine a common response in the two different cell lines, for example by comparing pathogenic stages (3 WPI) in Bm5 cells with late infection (24–36 HPI) in S2 cells, and persistent stages (2 DPI-1 WPI) in Bm5 cells with early infection (6–12 HPI) in S2 cells. Surprisingly, only limited similarity was found in the metabolic profile between the two infected cell lines during the course of infection and in relation to uninfected cells. Thus, although it has been reported that viruses belonging to different families could cause some common metabolic responses [23,24], in our study it was observed that CrPV could induce only minimal common metabolic changes in different host cells.

On the other hand, the metabolic differences between different host cells were considered an important observation since they provide an (partial) explanation for the cell-specific outcome of the virus infection. Metabolic pathway analysis revealed the importance of amino acid metabolism for the progression of CrPV infection in both cell lines (Table 3; Figure 8). Amino acids show an inverted pattern of dynamical changes between Bm5 and S2 cells, indicating that central carbon metabolism plays an important role during CrPV infection.

In CrPV-infected Bm5 cells, the pattern of change in amino acids was interpreted as compatible with an increase in protein building blocks (amino acids) during early (persistent) stages (2 DPI and 1 WPI) and their incorporation in viral proteins during high rates of virion production at late stages (2 WPI and 3 WPI) [13]. By contrast, our analysis indicated that such restriction did not exist for CrPV infection of S2 cells and that the decrease in amino acids and carbohydrates during early stages (6 HPI and 12 HPI; Figure 6 and Figure 7) might already reflect the consumption of building blocks for high levels of viral replication and virion reproduction. The increase in amino acids and main carbohydrates at late stages in S2 cells (Figure 8) might be a result of the degradation of the cellular organization caused by damage of viral replication and virion production, resulting in the release of proteases and hydrolases that break down proteins and other macromolecules.

Among all metabolites, both glucose and glutamine display the most dynamic changes during the course of infection of Bm5 cells with CrPV, with significant increases during early-middle infection followed by a sharp decrease at 2 and 3 weeks (Figure 8). However, the profile of glucose and glutamine is unique to Bm5 cells and was not observed during the short-duration pathogenic infection of S2 cells (Figure 6 and Figure 7). Notably, in S2 cells, opposite kinetics in abundances of sugars and amino acids were observed compared to Bm5 cells and the depletion of glucose and glutamine at the late stages was not detected. Both glucose and glutamine are major carbon sources to support energetic and biosynthetic requirements [23] and their depletion was considered an important contributing factor to cell death during pathogenic phases of CrPV infection in Bm5 cells [13]. In S2 cells, on the other hand, glucose as well as glutamine increased at 36 HPI (Table S1 and Figure 7) and therefore do not seem to be related to pathogenesis by CrPV.

In the baculovirus expression vector system (BEVS), the metabolic state of the cells at the start of infection is considered very important together with the ability of the virus to manipulate energy needs [25]. Of relevance may be the observation that at high cell densities the performance of BEVS is inhibited due to the suppression of central carbon metabolism [26]. Thus, the above results support the idea that CrPV depends on and can alter specific metabolic processes in different host cells and, consequently, that these virally induced alternations of host metabolism can result in different types of viral infection (persistence versus pathogenesis). However, in order to link the host specific metabolic changes to viral pathogenesis, it will be necessary to conduct further studies in different host cells where comparable infection kinetics are observed. For example, although S2 cells are more sensitive than the other cell lines that were investigated, the kinetics of infection in Hi5 cells can be considerably accelerated after application of CrPV at much higher MOI [11]. Thus, comparative metabolomics studies could be extended between pathogenically infected S2 and Hi5 cells as well as among several other persistently infected lepidopteran and coleopteran cell lines.

Our study also highlights changes in metabolism that are common with virus infections in other systems. In several mammalian virus infection models, both glucose and glutamine are required for optimal viral replication (e.g., for cytomegalovirus, herpex simplex virus and poliomyelitis virus; [27,28,29]) while only glutamine was important for vaccinia virus infection [30]. As mentioned above, both glucose and glutamine may have important roles during CrPV infection of Bm5 cells while they do not show major changes in abundance during the infection of S2 cells.

While characteristic changes in the abundance of polyamines were observed during CrPV infection of both Bm5 and S2 cells, a recent study also showed that polyamines are essential for the replication of two other RNA viruses, chikungunya virus (CHIKV) and Zika virus (ZIKV) [31]. Furthermore, polyamine depletion following induction of a catabolic enzyme by type I interferon was demonstrated as an antiviral strategy to restrict CHIKV and ZIKV replication. Our study indicated that the CrPV infection is also regulated by polyamines, which can be confirmed in functional experiments employing polyamine synthesis blockers and corresponding rescue with exogenous polyamines.

Acetylcarnitine shows a precipitous decline during the pathogenic stages of CrPV infection of S2 (but not Bm5) cells. Interestingly, elevated levels of acylcarnitines (although not acetylcarnitine) were observed during infection of mosquito midguts with the Dengue virus [32]. Since infections of CrPV in Bm5 cells and Dengue virus in mosquito midguts have a persistent character, our data may indicate the importance of the regulation of transport of fatty acyl-CoA from the cytoplasm to mitochondria to maintain the persistent state of the virus infection.

## 5. Conclusions

In summary, metabolomics was employed on CrPV persistently infected Bm5 cells and acutely infected S2 cells, to investigate how CrPV manipulates Bm5 metabolism for its persistence, and additionally, how those viral-induced metabolic changes may affect CrPV pathogenesis. Our data revealed important changes in host metabolism during CrPV persistence and pathogenesis and identified several metabolic characteristics during CrPV infection of Bm5 and S2 cells. While amino acids play an important role during CrPV infection, our analysis suggests that their levels were sufficiently high in S2 cells to trigger acute infection. In Bm5 cells, on the other hand, amino acid synthesis may need to be activated, which may explain the period of limited CrPV replication (persistence) that occurs in this cell line. Similarly, significant modulations of the levels of glucose and glutamine, related to energy production, were observed in Bm5 cells but not in S2 cells. In addition, polyamines seem to be critical for CrPV persistence and pathogenesis. Further functional analysis is needed to confirm requirements of specific metabolites for optimal CrPV replication, and additional experiments are required to identify unknown components from the untargeted metabolomics analysis to reveal important host factors during CrPV infection. Finally, integration of the metabolomics approach with other omics approaches [33,34] is necessary to advance our understanding of the process of persistence and pathogenesis in insect cell lines.

## Figures and Tables

**Table 1:** Classification dataset composition, and specification of constructed OPLS-DA models with the output of the model validation. Dataset includes both positive and negative ionization mode.

Model	Total Number of Instances	Number of Model Components (t_o_ + t_p_) ^a^	Model Characteristics ^b^	Cross-Validated ANOVA ^c^ (*p*-Value)	Permutation ^d^
6 HPI vs. 0 HPI	10	1+0	R^2^Y = 0.998; Q^2^ = 0.995	1.25 × 10^−8^	Good
12 HPI vs. 0 HPI	10	1+0	R^2^Y = 1.000; Q^2^ = 0.998	1.98 × 10^−10^	Good
24 HPI vs. 0 HPI	10	1+0	R^2^Y = 0.998; Q^2^ = 0.993	2.34 × 10^−8^	Good
36 HPI vs. 0 HPI	10	1+0	R^2^Y = 0.997; Q^2^ = 0.992	5.29 × 10^−8^	Good
12 HPI vs. 6 HPI	10	1+1	R^2^Y = 0.985; Q^2^ = 0.940	3.23 × 10^−3^	Good
24 HPI vs. 6 HPI	10	1+1	R^2^Y = 0.997; Q^2^ = 0.990	3.42 × 10^−5^	Good
36 HPI vs. 6 HPI	10	1+1	R^2^Y = 0.998; Q^2^ = 0.997	1.94 × 10^−6^	Good
24 HPI vs. 12 HPI	10	1+0	R^2^Y = 0.998; Q^2^ = 0.993	2.46 × 10^−8^	Good
36 HPI vs. 12 HPI	10	1+0	R^2^Y = 0.993; Q ^2^= 0.99	8.96 × 10^−8^	Good
36 HPI vs. 24 HPI	10	1+1	R^2^Y = 0.995; Q^2^ = 0.986	8.63 × 10^−5^	Good

**^a^** with t_o_ the orthogonal and t_p_ the predictive component; **^b^** with R^2^Y the variation in Y that is explained by the model, and Q^2^ the predictive ability of the model. Q^2^ > 0.5 indicates good model quality; **^c^** a cross-validated ANOVA *p*-value < 0.05 indicates good model quality; **^d^** good permutation testing is achieved if R^2^Y and Q^2^ values of the models based on the permutated data are significantly lower than those based on the real data set. “Q^2^ > 0.5”, “*p*-value < 0.05” and “good permutation test” mean that OPLS-DA can successfully separate comparing groups.

**Table 2:** Major metabolic pathways modulated during CrPV infection of S2 cells.

Comparison	Pathway	Hits	Holm Adjusted P	FDR	Impact
6 HPI vs. 0 HPI	None				
12 HPI vs. 0 HPI	Galactose metabolism	5	5.37 × 10^−3^	4.28 × 10^−3^	0.18
	Aminoacyl-tRNA biosynthesis	7	8.45 × 10^−3^	4.28 × 10^−3^	0.00
	Starch and sucrose metabolism	4	1.39 × 10^−2^	4.77 × 10^−3^	0.21
24 HPI vs. 0 HPI	None				
36 HPI vs. 0 HPI	Arginine and proline metabolism	7	8.43 × 10^−4^	8.43 × 10^−4^	0.33
	Aminoacyl-tRNA biosynthesis	8	5.93 × 10^−3^	3.00 × 10^−3^	0.14
	Alanine, aspartate and glutamate metabolism	4	1.23 × 10^−1^	4.19 × 10^−2^	0.61
12 HPI vs. 6 HPI	None				
24 HPI vs. 12 HPI	Aminoacyl-tRNA biosynthesis	9	1.05 × 10^−4^	1.05 × 10^−4^	0.14
	Galactose metabolism	5	6.95 × 10^−3^	3.52 × 10^−3^	0.18
	Starch and sucrose metabolism	4	1.72 × 10^−2^	5.90 × 10^−3^	0.21
	Arginine and proline metabolism	5	3.90 × 10^−2^	1.01 × 10^−2^	0.29
	Alanine, aspartate and glutamate metabolism	4	5.78 × 10^−2^	1.22 × 10^−3^	0.61
36 HPI vs. 24 HPI	Aminoacyl-tRNA biosynthesis	8	1.95 × 10^−3^	1.95 × 10^−3^	0.14
	Arginine and proline metabolism	5	5.12 × 10^−2^	2.59 × 10^−2^	0.33

Pairwise metabolite pathway analysis was conducted on the identified metabolite relative abundances between different time points after CrPV infection. Pathways with the most hits, highest Holm adjusted *p*-value (FDR < 0.05) and highest pathway impact values are considered the most significantly affected pathways. Hits refer to the number of metabolites that match to a particular metabolic pathway. The metabolic pathways with impact values > 0.1 are considered the most relevant pathways in the conditions under study.

**Table 3:** Summary of significant changes in abundance of amino acids and carbohydrates during CrPV infection in Bm5 and S2 cells (compared to uninfected cells).

		Amino acids	Carbohydrates	All Metabolites
**Bm5 cells**	2 DPI (*N* = 34)	19↑ 4↓	5↑	28↑ 6↓
	1 WPI (*N* = 45)	25↑ 1↓	9↑	41↑ 4↓
	2 WPI (*N* = 42)	21↑ 5↓	6↑ 1↓	32↑ 10↓
	3 WPI (*N* = 43)	20↑ 6↓	7↑ 1↓	32↑ 11↓
**S2 cells**	6 HPI (*N* = 13)	1↑ 4↓	3↑ 2↓	5↑ 8↓
	12 HPI (*N* = 21)	1↑ 9↓	3↑ 5↓	4↑ 17↓
	24 HPI (*N* = 6)	4↑ 1↓		4↑ 1↓
	36 HPI (*N* = 24)	10↑ 4↓	2↑ 3↓	14↑ 10↓

## Supplementary Materials

The following are available online at https://www.mdpi.com/1999-4915/12/4/393/s1, Figure S1: Figure S1: Heat maps of abundance of metabolites at different time points after CrPV infection: comparison with uninfected cells, Figure S2: Figure S2: Volcano plots of all identified metabolites in S2 cells at different time points of CrPV infection, Table S1: Table S1. Peak abundance ratio of identified metabolites in CrPV-infected S2 cells.
