# Supplementary material for: Metabolomic Analysis of Cricket paralysis virus Infection in Drosophila S2 Cells Reveals Divergent Effects on Central Carbon Metabolism as Compared with Silkworm Bm5 Cells
Source: Viruses. 2020 Apr 1;12(4):393. doi: 10.3390/v12040393 (PMC7232303; doi:10.3390/v12040393)
Supplement: Supplementary file 1 [file viruses-12-00393-s001.pdf]

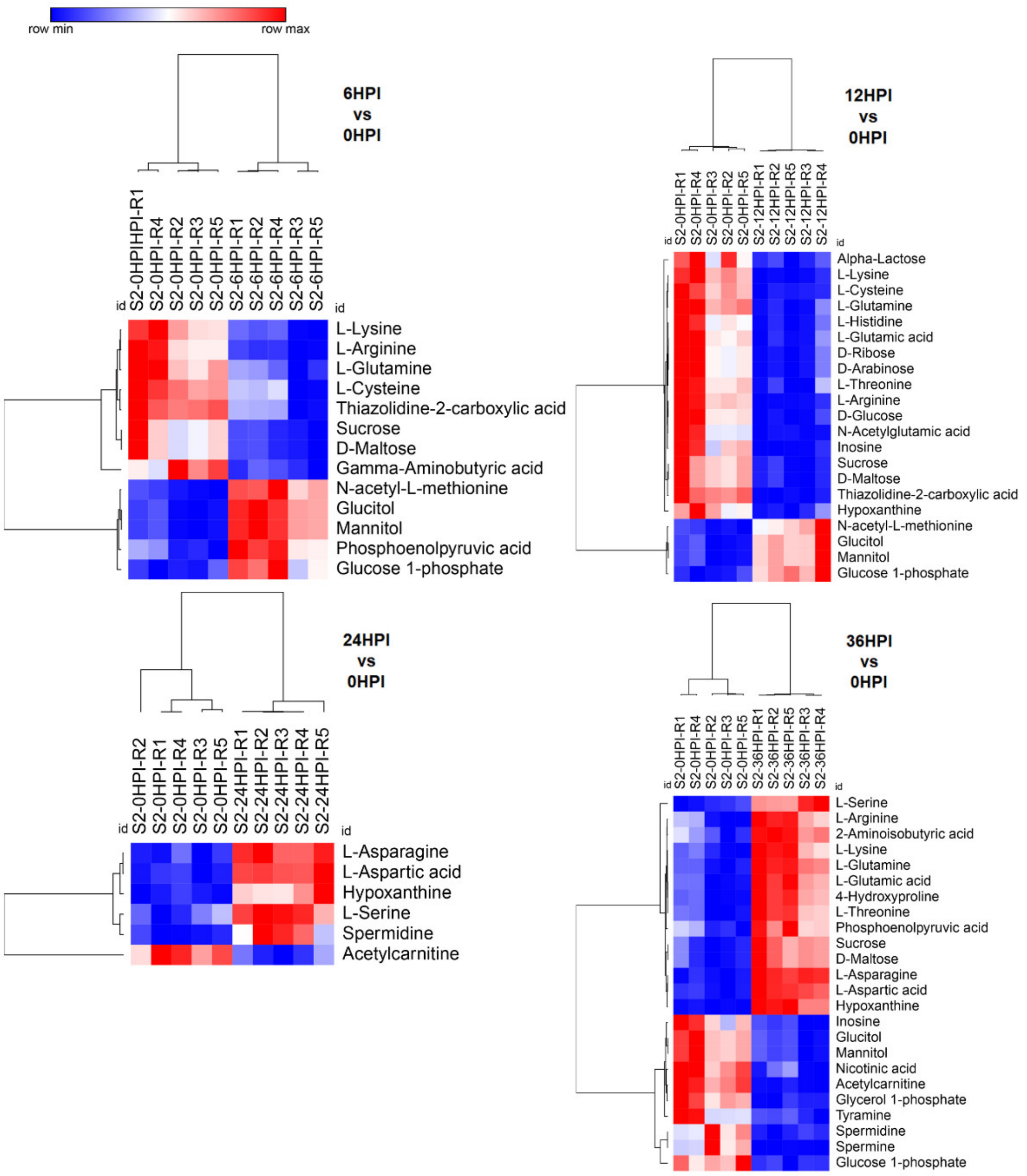

Figure S1: Heat maps of abundance of metabolites at different time points after CrPV infection: comparison with uninfected cells. Hierarchical clustering (one minus Pearson correlation) was used to separate individual samples (X-axis). The Y-axis represents individual metabolites that were identified and showed changes in abundance with respect to preceding and following time points ($p < 0.05$). Normalized signal intensities are visualized as a color spectrum in the heat maps. Red and blue represent higher and lower expression, respectively, of metabolites.

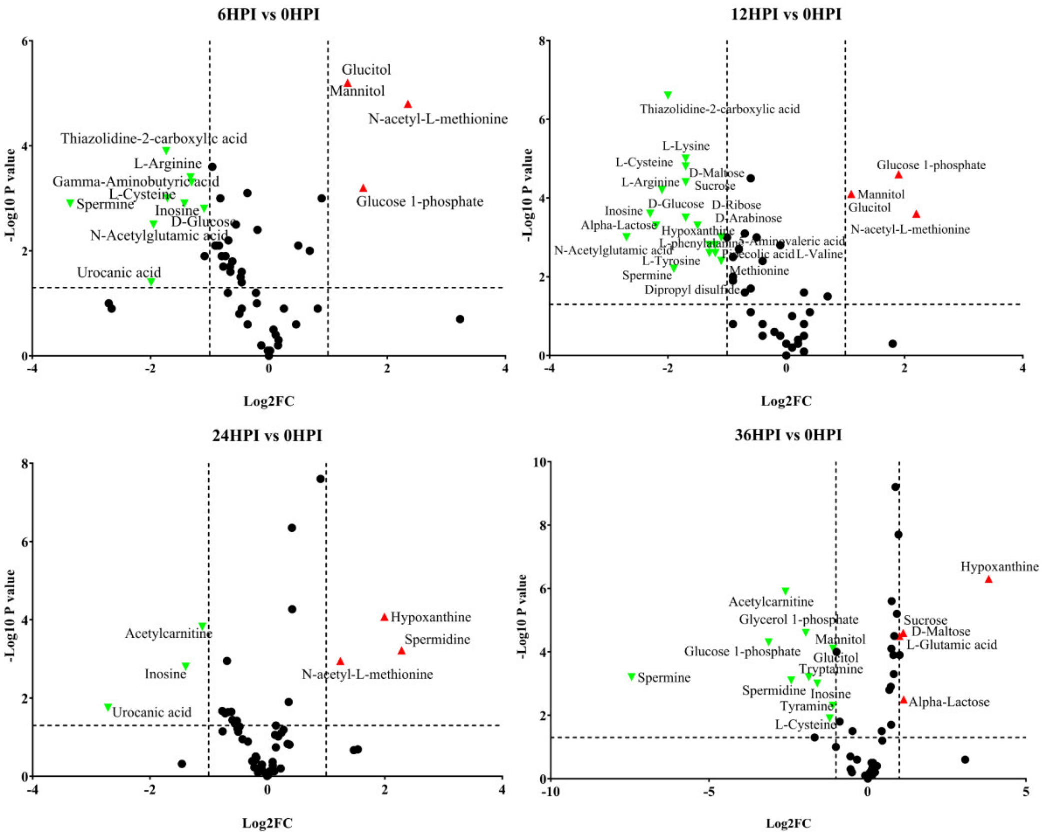

Figure S2: Volcano plots of all identified metabolites in S2 cells at different time points of CrPV infection: comparison with uninfected cells. In the volcano plot, the log scaled value of the fold change (FC, *x*-axis) is plotted against the –log 10 *p*-value from multiple *t*-test analysis (*y*-axis). The dashed lines delineate metabolites with fold change > 2 and *p*-value < 0.05. Compound identities are displayed for both upregulated (right side; red upward triangle) or downregulated (left side; green downward triangle) metabolites.

Table S1. Peak abundance ratio of identified metabolites in CrPV-infected S2 cells. Red and blue colors represent a significant ($p < 0.05$) increase and decrease in abundance, respectively (compared to uninfected cells). Values represent normalized abundance levels for each metabolite.

	6 hours	12 hours	24 hours	36 hours
Amino Acids				
L-Arginine	0.54	0.32	1.25	2.38
L-Asparagine	0.87	0.79	1.16	1.60
L-Aspartic acid	0.71	0.88	1.53	1.60
L-Cysteine	0.90	0.94	3.21	1.30
L-Glutamic acid	0.71	0.63	1.33	2.08
L-Glutamine	0.82	0.68	1.17	1.80
L-Histidine	0.89	0.63	1.19	1.96
L-Isoleucine	0.96	0.71	1.18	1.79
L-Leucine	0.96	0.71	1.18	1.79
L-Lysine	0.63	0.39	1.26	2.48
L-Methionine	1.01	0.74	1.12	1.70
L-phenylalanine	1.04	0.73	1.13	1.76
L-Proline	0.97	0.73	1.16	1.82
L-Serine	0.88	0.81	1.11	1.58
L-Threonine	0.93	0.73	1.18	1.80
L-Tyrosine	1.08	0.75	1.12	1.75
L-Valine	0.96	0.70	1.15	1.78
N-Acetyl-Methionine	2.62	2.36	1.22	0.86
N-Acetyl-Glutamic acid	0.56	0.34	1.28	2.68
L-Pyro-Glutamic acid	0.81	0.84	1.32	1.75
4-Hydroxy-Proline	0.90	0.77	1.19	1.88
Carbohydrates				
D-Arabinose	1.01	0.71	1.08	1.70
D-Fructose	0.45	3.62	8.26	0.00
D-Galactose	0.45	3.67	8.41	2.06
D-Glucose	0.79	0.53	1.10	1.86
D-Maltose	0.57	0.31	1.22	2.20
D-Ribose	1.01	0.71	1.08	1.70
Alpha-lactose	0.49	0.22	1.31	2.27
Glucitol	2.14	1.83	0.90	0.40
Mannitol	2.14	1.83	0.90	0.40
Gluconic acid	1.30	1.07	1.06	1.23
Glucose-1-phosphate	2.16	2.71	0.63	0.08
Sucrose	0.57	0.31	1.22	2.20
Other metabolites				
Acetylcarnitine	1.96	1.67	0.64	0.23

2-Aminoisobutyric acid	0.67	0.62	1.14	1.89
4-Aminobutyric acid	0.67	1.09	1.41	0.82
5-Aminovaleric acid	0.96	0.70	1.15	1.78
Citric acid	1.30	1.33	1.51	1.61
Cyclohexylamine	1.56	1.33	1.13	1.26
Dipropyldisulfide	1.01	0.74	1.13	1.72
Glycaraldehyde	1.47	1.58	1.10	0.90
Glycerol	5.47	1.96	0.21	4.93
Glycerol-1-phosphate	1.86	1.46	1.16	0.30
L-Gulonic_Acid Gamma Lactone	2.21	0.96	1.12	1.46
Hypoxanthine	0.22	0.12	1.38	4.89
Imidazolepropionic acid	1.00	0.84	1.11	1.01
Inosine	1.31	0.72	1.34	1.17
3-Methyl-2-Cyclohexen-1-one	1.10	0.99	1.02	1.18
Nicotinic_acid	1.47	1.20	0.85	0.69
Pantothenic_acid	1.21	1.14	1.43	1.24
3-Phenylpropionic acid	0.99	1.04	1.09	0.98
Phospho(enol)pyruvic acid	1.94	1.41	0.98	1.74
Pipecolic_acid	0.93	0.75	1.14	1.77
Spermidine	0.88	1.62	4.71	0.18
Spermine	0.47	1.35	5.71	0.03
Thiazolidine-2-carboxylic acid	0.61	0.49	2.40	1.11
Tryptamine	1.38	1.30	1.45	0.54
Tyramine	1.41	1.02	1.13	0.90
Urocanic acid	0.35	0.74	0.21	0.44
